# News

**DOI:** 10.1155/S1463924600000183

**Published:** 2000


Journal of Automated Methods & Management in Chemistry, Vol. 22, No. 4 (July–August 2000) pp. 123–124

### News

### Lab automation: the dramatic impact of drug discovery

*Los Angeles, CA, 9 September 1999*—Strategic Directions International is pleased to announce the publication of its latest Market Analyses and Perspectives (MAP) research report entitled *Lab Automation: The Dramatic Impact of Drug Discovery*. This report provides a detailed picture of the current market for various laboratory automation techniques and includes an in-depth forecast till 2003. Laboratory automation products discussed in this report include microplate readers, microplate auxiliary equipment, diluters/dispensers, XYZ workstations, organic synthesizers, automated solid phase extraction apparatuses, robotic stackers/arms, integrated robotic platforms, and software.

Liquid handlers, the largest segment of the laboratory automation products included in this report, generated worldwide revenues of $465 million in 1998. Annual growth for this most important segment is forecasted at ~15% per year for the next 5 years, contributing to a total lab automation market of ~$2.1 billion by 2003. Driven by the pharmaceutical and biotechnology industries' focus on high-throughput screening and combinatorial chemistry methods, demand for liquid handlers has experienced renewed growth in recent years.

Although liquid handlers currently account for the largest segment of the lab automation market, the fastest growth is expected to come from software. Specifically, cheminformatics software and integrated automation software are anticipated to grow at over 20% per year for the next 5 years.

Owing to benefits such as higher throughput, fewer error-prone tasks, and more reproducible results, many industries find laboratory automation an attractive investment. Accordingly, this renewed interest in lab automation has put it on a new growth curve. The pharmaceutical industry will continue to be the largest and fastest growing sector with demand expected to exceed $700 million by 2003.

This report assesses the lab automation market by major product types including: microplate readers and auxiliary equipment, diluters/dispensers, XYZ workstations, organic synthesizers, automated solid-phase extraction apparatus, robotic stackers/arms, integrated robotic platforms, and software. Secondary screening instruments, e.g. high-performance liquid chromatography (HPLC), mass spectrometry (MS) and nuclear magnetic resonance spectroscopy (NMR) are also discussed and quantified in relation to their role in drug discovery applications. Additionally, the market is segmented by geographic region and industry. Included in the report are company profiles, competitive analyses and market shares, as well as end-user opinions. This 229 page report, containing 121 tables and 134 figures, provides a complete analysis of the market at the price of $3895.

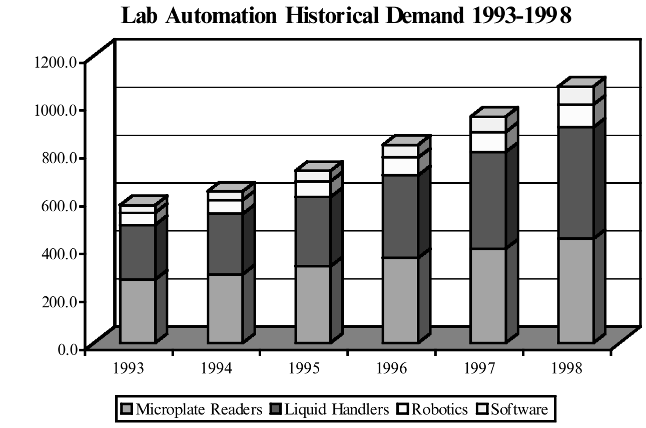

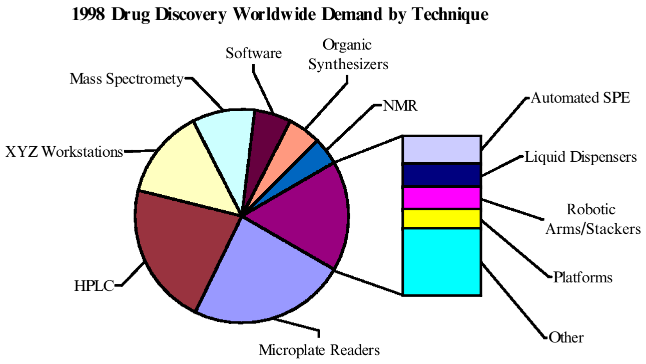

*For more information please contact Strategic Directions International, 6242 Westchester Parkway, Suite 100, Los Angeles, CA 90045, USA. Tel: + 1 (310) 641-4982; Fax: + 1 (310) 641-8851; e-mail: sdi@strategic-directions.com or Biote Postale 7, 78131 Les Mureaux Cedex, France. Tel: + 33 (1) 30 99 62 50; Fax: + 33 (1) 34 74 81 62; e-mail: sdi_europe@compuserve.com*

Strategic Directions International, based in Los Angeles, CA, is the leading international consulting company in the specialized field of analytical instrumentation. Our dedication to the field allows us to offer our clients a high level of detail and insight that comes from constantly monitoring the developments in the industry. News releases and other information about SDi are available at www.strategic-directions.com

### HP Launches the first product based on 'lab-on-a-chip' technology

*Geneva, Switzerland, 24 September 1999*—Hewlett-Packard Europe today introduced the HP 2100 bioanalyser for the integrated analysis of nucleic acids. This is the first commercially available product utilizing lab-on-a-chip technology.

Based on Caliper Technologies' LabChip™ technology, the system marks a major step toward the development of a completely automated microscale laboratory. In this 'lab-on-the-chip', all sample preparation, fluid handling and biochemical analysis steps will be carried out within the confines of a microchip. The chips comprise micro-channels that create interconnected networks of fluid reservoirs and pathways.

Used together with various LabChip kits, the HP 2100 bioanalyser improves the quality of nucleic acid analysis by integrating sample handling, separation, detection and digital data processing within a single, compact system architecture. The system is designed for use by molecular biologists and biochemists working with poly-merase chain reaction (PCR) products, restriction en-zyme digests or ribonucleic acid (RNA) preparations.

*Implications for life-science research*

Researchers in the areas of disease and drug discovery still experience manual processing bottlenecks during the analysis of nucleic acids. LabChip technology offers many benefits over the existing manual gel electrophor-esis products.

- *Improved accuracy.* The integrated, miniaturized chip design drastically cuts system retention and material transfer losses, improving accuracy and reducing sample consumption.
- *Rapid analysis.* Miniaturized fluid pathways dramatically shorten run times. Each nucleic acid sample is analysed in 90 s.
- *Enhanced reproducibility.* State-of-the-art fabrication processes yield chips that perform more repro-duc-ibly than conventional gel products. Automated fluid handling, using standardized experimental protocols, further enhances performance.

*Initial applications*

The HP 2100 bioanalyser is offered with three intro-ductory assays. Each assay is packaged as a complete, ready-to-use kit.

- DNA 7500 LabChip kit for accurate sizing and quantitation of DNA fragments ranging in size from 100 to 7500 bp.
- DNA 12000 LabChip kit for accurate sizing of DNA fragments from 100 to 12 000 bp.
- RNA 6000 LabChip kit for the analysis and quan-titation of total RNA and mRNA samples.

*Pricing and availability*

The HP 2100 bioanalyser is initially available in Germany, Switzerland and the USA. It can be ordered directly through HP chemical analysis products sales offices in each of these countries. The price of the complete system is approximately Euro 20 000 and delivery is estimated at 6 weeks from receipt of order. The system is expected to be available in additional European countries and Japan by the end of 2000.

*About HP's Chemical Analysis Group*

HP's Chemical Analysis Group, with headquarters in Palo Alto, CA, USA, is a leading provider of chemical analysis measurement and information solutions, services and support, columns and supplies to scientific labora-tories in industry, government and academia. The group has 3789 employees worldwide and had revenues of $946 million in its 1998 fiscal year.

Information about LabChip products, can be found on the World Wide Web at http://www.hp.com/go/labona-chip

*About HP*

Hewlett-Packard Company—a leading global provider of computing and imaging solutions and services for business and home—is focused on capitalising on the opportunities of the Internet and the proliferation of electronic services.

HP plans to launch Agilent Technologies as an indepen-dent company by mid-calendar year 2000. Agilent con-sists of HP's test and measurement, semiconductor products, chemical analysis and healthcare solutions businesses, and has leading positions in multiple market segments.

HP has 123 500 employees worldwide and had a total revenue of $47.1 billion in its 1998 fiscal year. Informa-tion about HP, its products and the company's Year 2000 program can be found on the World Wide Web at http://www.hp.com

LabChip™ and the LabChip logo are USA trademarks of Caliper Technologies Corporation.

Hewlett-Packard Company has introduced the HP 2100 bioanalyser for the integrated analysis of nucleic acids. This is the first commercially available product utilizing lab-on-a-chip technology.

*Please send reader enquiries to: Hewlett-Packard Europe, PO Box 165, Godalming, Surrey GU7 1WD, UK*

